# Diversity of lactase persistence in African milk drinkers

**DOI:** 10.1007/s00439-015-1573-2

**Published:** 2015-06-09

**Authors:** Bryony Leigh Jones, Tamiru Oljira, Anke Liebert, Pawel Zmarz, Nicolas Montalva, Ayele Tarekeyn, Rosemary Ekong, Mark G. Thomas, Endashaw Bekele, Neil Bradman, Dallas M. Swallow

**Affiliations:** Research Department of Genetics, Evolution and Environment, University College London, Darwin Building, Gower Street, London, WC1E 6BT UK; Department of Microbial Cellular and Molecular Biology, Addis Ababa University, Addis Ababa, Ethiopia; Department of Biology, Faculty of Natural and Computational Sciences, University of Haramaya, P.O. Box 32597, Addis Ababa, Ethiopia; Henry Stewart Group, 28/30 Little Russell Street, WC1A 2HN London, UK

## Abstract

The genetic trait of lactase persistence is attributable to allelic variants in an enhancer region upstream of the lactase gene, *LCT*. To date, five different functional alleles, −13910*T, −13907*G, −13915*G, −14009*G and −14010*C, have been identified. The co-occurrence of several of these alleles in Ethiopian lactose digesters leads to a pattern of sequence diversity characteristic of a ‘soft selective sweep’. Here we hypothesise that throughout Africa, where multiple functional alleles co-exist, the enhancer diversity will be greater in groups who are traditional milk drinkers than in non-milk drinkers, as the result of this sort of parallel selection. Samples from 23 distinct groups from 10 different countries were examined. Each group was classified ‘Yes ‘or ‘No’ for milk-drinking, and ethnicity, language spoken and geographic location were recorded. Predicted lactase persistence frequency and enhancer diversity were, as hypothesised, higher in the milk drinkers than the non-milk-drinkers, but this was almost entirely accounted for by the Afro-Asiatic language speaking peoples of east Africa. The other groups, including the ‘Nilo-Saharan language speaking’ milk-drinkers, show lower frequencies of LP and lower diversity, and there was a north-east to south-west decline in overall diversity. Amongst the Afro-Asiatic (Cushitic) language speaking Oromo, however, the geographic cline was not evident and the southern pastoralist Borana showed much higher LP frequency and enhancer diversity than the other groups. Together these results reflect the effects of parallel selection, the stochastic processes of the occurrence and spread of the mutations, and time depth of milk drinking tradition.

**Electronic supplementary material:**

The online version of this article (doi:10.1007/s00439-015-1573-2) contains supplementary material, which is available to authorized users.

## Introduction

The genetically determined trait of persistence of intestinal lactase into adult life, which allows adult consumption of milk without adverse side-effects, is attributable to allelic variants in a regulatory region upstream of the lactase gene, *LCT* (Ingram et al. 2009a). The ancestral state and most common phenotype worldwide is for lactase to be down-regulated during childhood—lactase non-persistence (Ingram et al. 2009a). Lactase persistence however is the most prevalent phenotype in Europeans, particularly in the North, but has also been shown to be widespread in East Africa and the Middle East where it has been associated with a pastoralist way of life. Indeed the first observations of this correlation, in the 1960–1980s, led to the hypothesis that the lactase persistence trait was under strong positive selection due to the benefits of milk drinking (Simoons 1970; Aoki 1986; Holden and Mace 1997).

To date, five different alleles, −13910*T, −13907*G, −13915*G, −14009*G and −14010*C, within a sequence 14,000 bp upstream of *LCT*, each associated with lactose digester status and occurring on different haplotypic backgrounds, have been shown to affect enhancer function in vitro, providing clear evidence that the LP trait has evolved several times independently (Enattah et al. 2002; Troelsen et al. 2003; Ingram et al. 2007; Tishkoff et al. 2007; Jones et al. 2013). The effect of this has been to cause the enhancer region to have much higher diversity in Ethiopian and Sudanese lactose digesters than non-digesters (Ingram et al. 2009b; Jones et al. 2013). By examining sequences flanking the enhancer we were able to show that there was very similar (and high) background haplotype diversity in digesters and non-digesters, implying that this differential enhancer diversity was not due to hidden population substructure associated with demographic effects, and could be most readily explained by the effect of selection on the functional alleles within the enhancer region (Jones et al. 2013). This pattern of diversity is characteristic of the effects of a soft selective sweep (Hermisson and Pennings 2005; Pennings and Hermisson 2006a, b), the phenomenon by which several alleles with similar effect on function are selected in parallel.

These results were in stark contrast to the high frequency of a single LP variant (−13910*T) and reduced haplotype diversity (Harvey et al. 1998; Hollox et al. 2001; Poulter et al. 2003; Bersaglieri et al. 2004; Gallego Romero et al. 2012) observed throughout Europe and some parts of central and southern Asia, and also the predominance of −14010*C on a single extended haplotype in Tanzania (Tishkoff et al. 2007), both of which show the classic pattern of selection of a hard selective sweep (Sabeti et al. 2002).

However in Africa overall, where multiple functional alleles co-exist, we might predict that the enhancer diversity would be greater in traditional milk drinking pastoralist groups than those who were non-pastoralists, as a result of the parallel selection of these functional alleles. Furthermore, by carefully examining the region showing that increased diversity we might uncover novel functional variants.

Since it is not possible to classify the groups collected simply as pastoralist or non-pastoralist, we instead used milk drinking as a ‘Yes’ or ‘No’ value obtained from the Murdock catalogues (Murdock 1959, 1967). In examining the causes of such a difference in genetic diversity it is important to consider the relative relatedness of the groups, and the possibility that there are large differences in overall diversity between peoples of such varied demographic origins whose effective population size, pattern of migration and reproductive behaviour are likely to be very different. By taking the same approach that we did for our recent study on lactose digesters and non-digesters, examining sequences ~16 kb upstream and ~13 kb downstream of the enhancer sequence (Jones et al. 2013), we controlled at least in part for such differences. Distributions were also examined geographically and groups classified by their language family.

## Methods

DNA samples from the collection currently stored in UCL for population genetics studies, and also for our studies on lactase persistence (Hollox et al. 2001; Ingram et al. 2007) (collected under Ethics UCLH 99/0196 and 01/0236 with associated local approvals) were from fully informed consenting anonymous volunteers selected to be as far as possible unrelated to the grandparental level. Samples were chosen for analysis with the aim of maximising homogeneity of self-declared ethnicity where this information was available: people with different self-declared ethnicities at the grandparental level were excluded from the study. The languages spoken were recorded and their classification into major language super-families and groups/subgroups is shown in Supplementary Table 1. Groups were classified for milk-drinking status using ethnographic resources (Murdock 1959, 1967). Populations that are described as primarily agricultural, with some milking of their animals, were conservatively classified as milk drinkers.

In addition to the main study, the *LCT* enhancer sequences of 5 extra Oromo groups were compared. These included four newly collected groups from different geographic regions. Data were obtained from 104 ‘Borana’ from Moyale in Southern Oromia on the Kenyan border, 88 Oromo from Fiche in Salale province, central Ethiopia, 88 from Harar in eastern Ethiopia and 86 from Begi in West Wallaga. Those in the south, the Borana Oromo, represent true pastoralists (Legesse 1973; Luseno et al. 1998; OCTB 2006), but all groups can be classified as milk-drinkers. We also compared the 74 Oromo described in our previous study, who were students at Haramaya University, but known to come from a variety of different home towns (Jones et al. 2013).

The *LCT* enhancer and flanking regions were sequenced on both strands by standard Sanger sequencing, as described previously (Jones et al. 2013). The coordinates on Ch 37/Hg 19 of the Human genome sequence of the regions included in this study were 136624997 to 136624667 for control region 1; 136608768 to 136608467 for the enhancer; 136595706 to 136595346 for control region 2. The regions at −16 kb and +13 kb were selected as control segments that had, because of their close linkage and limited recombination, followed a similar demographic history to the enhancer region (as discussed in Jones et al. 2013). The location of control region 1, also within *MCM6*, provides a region likely to have similar chromatin structure and thus exposure to mutation.

Alleles were phased using PHASE (Stephens et al. 2001) and diversity measures (Nucleotide diversity, Pi, haplotype heterozygosity, Nei’s H, as well as tests for departure from neutrality) calculated using DnaSP (Rozas 2009) (as described in Jones et al. 2013). Pairwise Fsts were calculated using Arlequin software (http://www.cmpg.unibe.ch/software/arlequin35) and PCO plots constructed as described previously (Veeramah et al. 2010). Permutation tests were done using the permutation spread-sheet created by Michael Wood (http://userweb.port.ac.uk/~woodm/nms/).

Contour maps were constructed by kernel density estimation as implemented in ‘R’ (v.3.0.2, 2013-09-25, “Frisby Sailing” for Mac-OS X 10.6 Snow Leopard) using the *spatstat* package (Baddeley and Turner 2005) and included weighting for sample size. Interpolation smoothing was conducted at the lowest non-overflowing bandwidth (value of sigma) allowable from the heterogeneous data available. Latitude and longitude of collection sites are given with approximate midpoints of group location taken where there were various or uncertain collection sites.

## Results

**Fig. 1:**
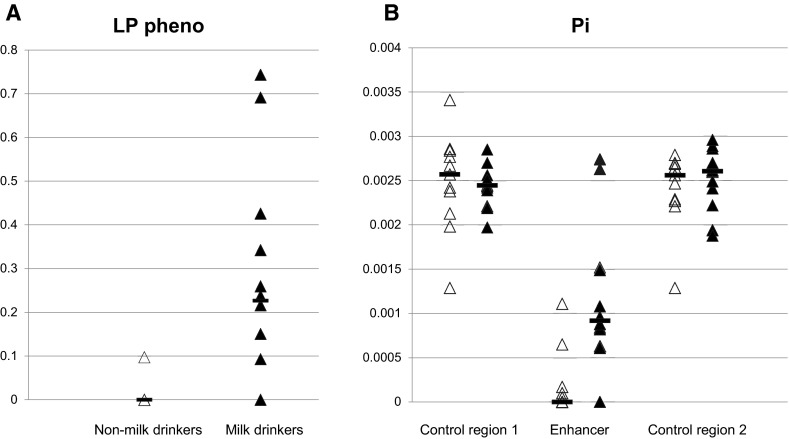
*Scatter plots* of lactase persistence (LP) and nucleotide diversity (Pi) in groups who traditionally milk animals and drink milk, or do not. **a** Inferred LP frequency in the milk drinking and non-milk drinking African groups. **b** Pi for the 3 regions sequenced. LP frequency calculated from the combined allele frequency using all 5 known functional alleles and assuming dominance, 2*pq* + *q* ^2^, where *q* = combined allele frequency for all 5 functional alleles and *p* corresponds to the combined allele frequency for all alleles without a functional variant. Milk drinkers *filled triangles*; non-milk drinkers *open triangles*. *Horizontal bars* show medians. Difference in LP frequency and difference in Enhancer Pi in milk drinkers and non-milk drinkers are both statistically significant (LP frequency: *p* = 0.0056; Enhancer Pi: *p* = 0.00288, Mann Whitney Rank test)

We collected data from 1061 samples from 23 distinct groups from 10 different countries over Africa (Table 1). In most cases they are defined by self-declared ethnic identity, as well as by geographic origin. Milk drinking status was classified as ‘Yes’ or ‘No’. Supplementary Table 1 shows the approximate geographic locations of the collection points. Enhancer alleles detected in each group are indicated in Table 1 while full allele frequencies for all three regions sequenced are shown in Supplementary Table 2. Lactase persistence frequency was inferred using the combined frequencies of the alleles shown to be functional in vitro and assuming dominance. LP frequency is on average greater in the milk drinkers than the non-milk drinkers (Fig. 1a). This difference is highly statistically significant (Mann–Whitney Rank test, *p* = 0.0056).

**Table 1:** Groups tested, showing their self-declared identity, collection location, animal milking and milk-drinking tradition, and *LCT* enhancer alleles detected

Country	Ethnic group	Linguistic super-family	Milk yes/no-species	Enhancer Nei’s H	Enhancer alleles detected
**West and Central Africa**					
Cameroon	Mambila	Niger-Congo	No	0.016	−13937*A
	Shuwa Arabs	Afro-Asiatic	Yes-cow^b^	0.184	**−** 13910*T, −13913*C, −13915*G
	Pygmy	Niger-Congo	No^a^	0.325	−13752*T, −13732*A
Congo	Bantu speakers: Kongo and other, collected Brazzaville	Niger-Congo	No	0	−
Ghana	Asante	Niger-Congo	No	0.029	−13732*A
	Builsa	Niger-Congo	No	0	−
Senegal	Mandjak	Niger-Congo	No	0	−
	Wolof	Niger-Congo	Yes-cow sheep, goat	0	−
**East Africa**					
Ethiopia	Afar	Afro-Asiatic	Yes-camel, cow, goat, sheep^b^	0.649	−13806*G, **−** 13907*G, **−** 13913*C, −13915*G , 13957*G, **−** 14009*G
	Amhara	Afro-Asiatic	Yes-cow, sheep, goat	0.305	−13779*C, −13806*G, **−** 13907*G, **−** 13913*C, **−** 13915*G, **−** 14009*G
	Anuak	Nilo-Saharan	Yes-cow, goat, sheep	0.184	−13800*T
	Maale	Afro-Asiatic	Yes-cow, goat, sheep	0.242	−13800*T, −13806*G, −13907*G, **−** 13915*G, **−** 14009*G
	Manjo	Afro-Asiatic	No^a^	0.19	−13753*T, **−** 13907*G, **−** 13913*C, **−** 14009*G
	Nuer	Nilo-Saharan	Yes-cow, goat, sheep^b^	0.243	−13753*T, −13800*T
	Oromo	Afro-Asiatic	Yes-cow, goat, sheep, sometimes camels^**c**^	0.407	−13800*T, −13806*G, −13907*G, −13913*C, **−** 13915*G, **−** 14009*G, **−** 14010*C
	Shabo	Nilo-Saharan?	No^a^	0.05	−
	Suri	Nilo-Saharan	Yes-cow	0.257	−13800*T, **−** 14010*C
Sudan	Beni Amer	Afro-Asiatic	Yes^**c**^ **-**sheep, goat, cow, camel	0.666	−13752*T, −13907*G, −13910*T, **−** 13915*G, −14009*G
	Jaali	Afro-Asiatic	Yes-cow	0.423	−13800*T, −13907*G, −13910*T, **−** 13913*C, **−** 13915*G, −14009*G
Tanzania	Chagga	Niger-Congo	Yes-cow, goat, sheep	0.283	−13800*T, **−** 14010*C, −13937*A
**Southern Africa**					
Mozambique	Bantu speakers: collected Sena	Niger-Congo	No	0	−
Malawi	Chewa	Niger-Congo	No	0	−
Namibia	San	Khoisan	No	0	−

All derived enhancer alleles detected are shown, with the confirmed functional alleles indicated in bold. (see Supplementary Tables 1 and 2 for language group and further details)

^a^Traditional hunter gatherer/hunter

^b^Herder/pastoralist

^c^Some are pastoralists

Haplotypes generated by PHASE were used to calculate the diversity measures, and to conduct tests for departure from neutrality. Although the standard sequence-based tests (Tajima’s D, Fu and Li’s D and F, and Fu’s Fs statistic) did not show statistical significance for departure from neutrality (data not shown), direct examination of the patterns of diversity showed clear differences between milk drinkers and non-milk drinkers. For the flanking sequences, Nei’s H and Pi showed very little difference across groups, and no difference in distribution between the milk and non-milk drinkers. However, the enhancer region was quite different (Table 1, Supplementary Table 2, Fig. 1b). This showed very little diversity in the non-milk drinkers but much greater diversity in the milk drinkers with a median Pi of 9.20E−04 (mean 1.20E−03) for milk drinkers compared with <1.00E−04 for non-milk-drinkers (mean 1.90E−04) (Fig. 1b). This difference in the enhancer was highly significant as assessed both by a Mann–Whitney test (*p* = 0.003) and by a permutation test (200 permutations, *p* < 0.005).

Examination of the enhancer diversity by a sliding window approach (Supplementary Figure 1) showed that the difference is almost entirely attributable to the two sequence regions at −13907 to −13915 and −14009/10, which house the alleles so far reported to be functional (Troelsen et al. 2003; Ingram et al. 2007; Tishkoff et al. 2007; Jensen et al. 2011; Jones et al. 2013). The variable region nearer to the *LCT* gene (−13732 to −13806) is less different between milk drinkers and non-milk drinkers. Of the variants detected, −13806*G was only found in Ethiopian milk drinkers but was shown in our recent study to be less frequent in digesters than non-digesters (Jones et al. 2013). The variant −13800*T was on the other hand more widespread and is more frequent in milk drinkers, but there was only one person (a non-digester) carrying −13800*T in our previous study on phenotyped individuals. This allele was interestingly mostly confined to Nilo-Saharan language speaking groups in this study. The very rare allele −13779*C was present in Amhara only in this data-set and −13752*T and −13753*T were both found in people from milk-drinking and non-milking groups, while −13732*A was found in two non-milk drinking groups.

**Fig. 2:**
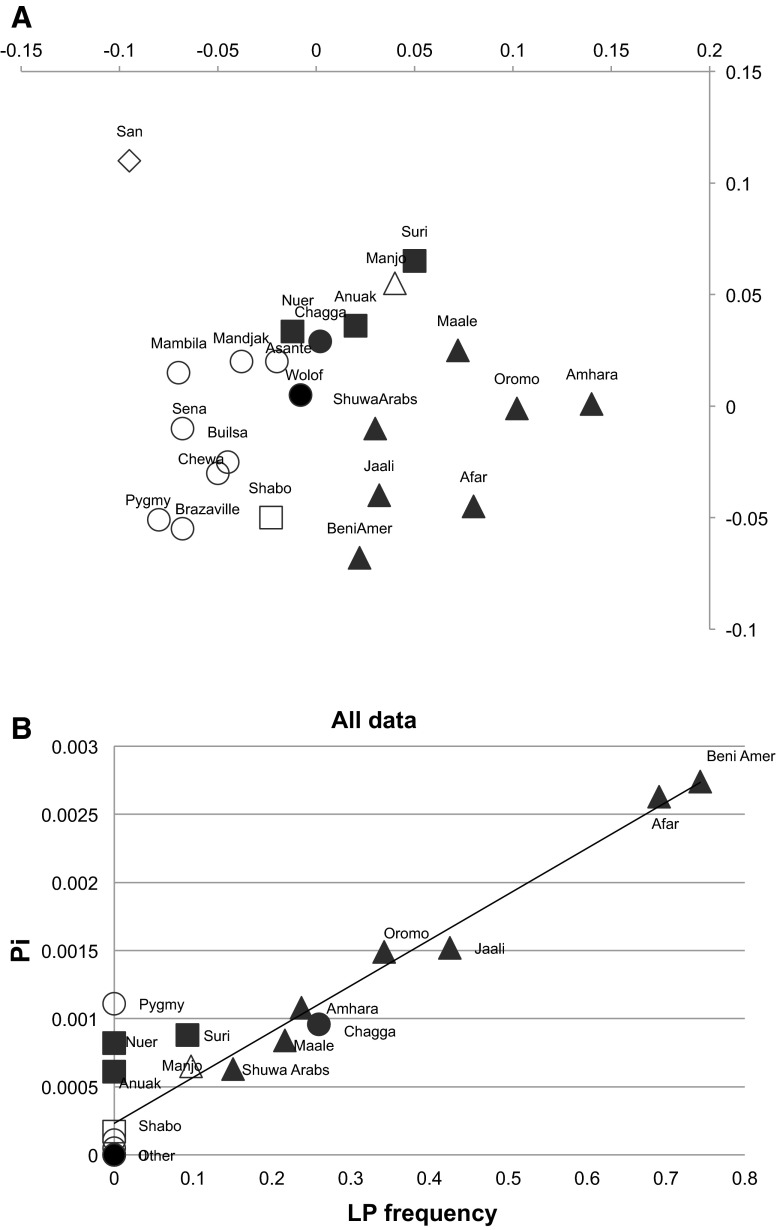
Genetic and linguistic differentiation of the groups under study. **a** PCO plot of pairwise Fsts showing the first two principal components, PCO1 (64.8 %) on the *X* axis and PCO2 (32.55 %) on the *Y* axis **b** Plot of nucleotide diversity, Pi (*Y* axis), versus LP frequency calculated from the combined data of all 5 functional alleles (*X* axis) *r* ^2^ = 0.86 *p* ≪ 0.001. Fsts calculated using the three sequenced regions combined, but similar results were obtained for the separated regions. *Triangles* Afro-asiatic language speakers; *circles* Niger Congo languages; *squares* Nilo-Saharan and diamond Khoisan. *Filled symbols* are milk-drinking populations while *open symbols* are not. Supplementary Figure 2 shows correlations of the data subdivided by language group and milk drinking status

Principal coordinates (PCO) plots of pairwise Fsts (Fig. 2a) show partitioning of the non-milk drinkers in comparison with the milk drinkers. However Niger-Congo language speaking groups (mainly Bantu language-speakers), shown as circles, are over-represented among the non-milk drinking groups while Afro-Asiatic language speakers (triangles) are over-represented among the milk drinking groups, showing the genetic differentiation of these two linguistic and life-style groups. Figure 2b shows the comparison of LP frequency and enhancer diversity of the Afro-Asiatic, Niger Congo and Nilo-Saharan groups.

**Fig. 3:**
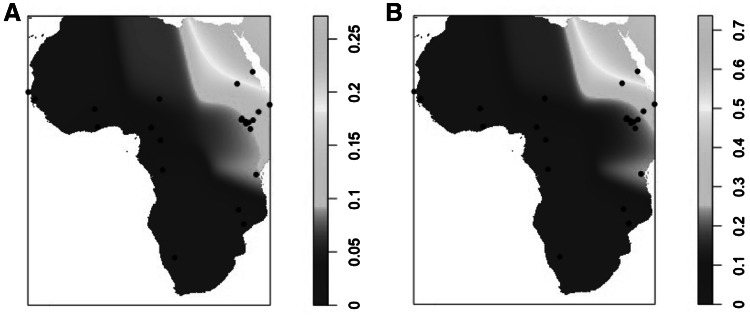
Interpolated smoothed contour maps of *LCT* enhancer diversity. **a** Pi × 10^2^ and **b** inferred lactase persistence frequency in Africa. *Dots* indicate data points. Lactase persistence frequency calculated from the sum of the five functional alleles (−13907*G, −13910*T, −13915*G, −14009*G and −14010*C), and assuming dominance

There is also a clear geographic cline in *LCT* enhancer diversity. Figure 3a shows a contour map depicting the strong north-east to south-west cline of diversity across Africa, compared with a similar plot in which LP frequency calculated by combining all five alleles is shown (Fig. 3b). The maps are almost identical: diversity is positively correlated with LP frequency across Africa, as estimated from combined allele frequency (Fig. 2b, *r*^2^ = 0.86, *p* ≪ 0.001). This correlation between Pi and LP stays significant when analysing groups separately according to linguistic family (Afro-asiatic populations, *r*^2^ = 0.99, *p* < 0.01, and non Afro-Asiatic populations, *r*^2^ = 0.28, *p* < 0.05), and also when analysing milk-drinkers only (*r*^2^ = 0.91, *p* < 0.001). The only group in which there is no significant correlation of Pi and LP is the non-milk drinkers (*r*^2^ = 0.19, *p* > 0.1). (See Supplementary Figure 2).

Maps of the geographic distributions of the individual alleles are shown in Supplementary Figure 3.

### Oromo groups with differing life-styles

**Fig. 4:**
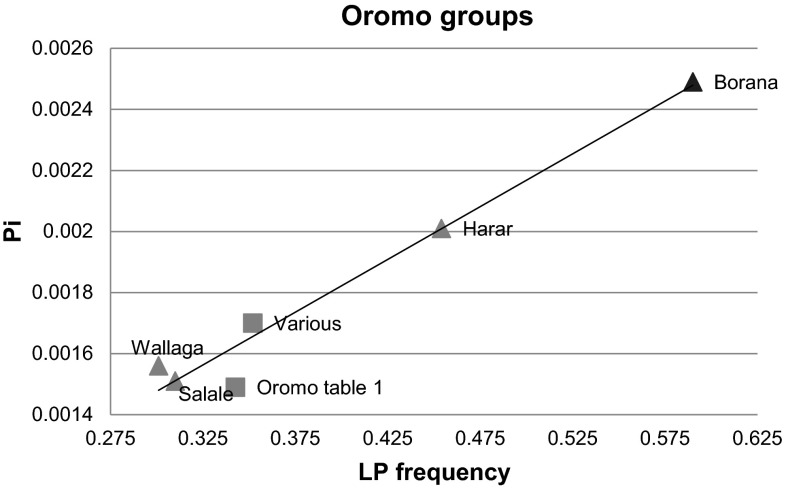
*Scatter plot* showing correlation of nucleotide diversity (Pi) (*Y* axis) and inferred LP frequency (*X* axis) in different Oromo groups. This strong correlation *r* ^2^ 0.98 *p* ≪ 0.001 is attributable to co-selection of 5 different LP alleles. 4 newly collected, geographically distinct groups are shown as *triangles*, (see Supplementary Table 3 for data) in comparison with the Oromo collection shown in Table 1, (‘Oromo table 1’), and also the Oromo group previously published (Jones et al. 2013) (‘Various’), both of which are shown as *squares*. The data point marked as a *darker triangle* is that obtained from the pastoralist Borana from the south

Since the difference in LP frequency and enhancer diversity in milk drinkers and non-milk drinkers is geographically structured, and is so confounded by difference in ancestry, we examined groups of more similar ethnic background, but whose lifestyle is different. We sequenced the enhancer region in 4 new groups of Oromo collected from distinct geographic regions (see Supplementary Table 1 and 3) that comprise one group of pastoralists, and 3 groups of agriculturalists who keep cows and drink milk. We compared these with the Oromo group described above and with the previously collected data from Oromo students, whose geographic origins were scattered (Jones et al. 2013). Again the *LCT* enhancer diversity is significantly correlated with inferred LP frequency *r*^2^ = 0.98 *p* ≪ 0.001 (Fig. 4), which is presumably attributable to the parallel selection of 5 different LP alleles in these related groups: the true pastoralists. The Borana, who are much more milk-dependent than the other groups, show by far the highest LP frequency and also *LCT* enhancer diversity. Notably, the same geographic cline in *LCT* diversity is not detected (Supplementary Table 1), with the most southerly group, the Borana, showing the highest diversity.

## Discussion

In this paper we show stark differences in both predicted lactase persistence frequency and *LCT* enhancer diversity between milk drinkers and non-milk drinkers. Milk-drinkers have almost tenfold greater nucleotide diversity for the enhancer region than the non-milk drinkers. There is however no difference in terms of allelic diversity in the other two segments sequenced between milk drinkers and non-milk drinkers, or between groups classified according to the major language affiliation. This suggests that no major demographic differences, such as caused by migration or differences in population size, account for the enhancer difference; this diversity difference therefore probably reflects the positive parallel selection we noted before (Jones et al. 2013), a signal associated with maintenance of haplotype diversity, which is not easy to detect using formal tests for departure from neutrality (Chevin et al. 2008). Here however the situation is also complicated by the fact that the enhancer sequence is notably lacking in diversity in lactose non-digesters and in non-milk drinkers. The fact that the enhancer (between positions −14028 and −13800) is rather conserved across primates (~93.5 % identity between humans, chimpanzee, gorilla, orangutan, gibbon, baboon and rhesus macaque (Jones et al. 2013, Figure S4)) and more so than its immediate flanking sequences, suggested purifying selection of this functional region, and we propose that these constraints are relaxed by life-style changes associated with milk consumption: i.e. the benefits of adult expression in people with this new life style outweighed any negative impact on childhood expression. However, we currently cannot exclude the contribution of additional effects that might have influenced his pattern of diversity, and we are investigating this further.

Despite the fact that examination of the linked control regions failed to show differences in diversity across groups there is clearly some population stratification in the sense that the milk drinkers in this study may be more related to each other than they are to the non-milk drinkers. While the 23 populations sampled were each of different self-declared cultural identity and/or geographic location and are linguistically diverse, there is a strong north-east to south-west cline in the pattern of enhancer diversity with the highest diversity in this data-set being in Sudan. In addition, while most of the Niger-Congo language speaking groups were non-milk drinking, most of the milk drinking groups speak Afro-Asiatic languages. Perhaps more importantly the groups with greatest enhancer diversity are all Afro-Asiatic language speakers; the Niger-Congo and Nilo-Saharan milk drinkers do not show greatly increased *LCT* enhancer diversity. The other ancestry related difference is the frequency of −14010*C that is very rare in the Afro-Asiatic groups, which is in contrast to the other functional alleles. In our data set it is most prevalent in a Niger-Congo language speaking group, but also present occasionally in the Nilo-Saharan Suri.

In Ethiopia and Sudan, the functional alleles seem to have spread across milk drinking groups, as might be expected from the movements of pastoralists, and positive selection for lactase persistence. Here, positive selection is best illustrated by the correlation of nucleotide diversity and inferred LP frequency in the Oromo groups and the particularly high enhancer diversity in the more milk dependent pastoralist Oromo.

From inspection of the Supplementary data of a previous major African study (Ranciaro et al. 2014) it can be seen that there is similar *LCT* enhancer diversity in Kenyan milk drinkers, with up to three of the known functional alleles being detected in one group, but this was not the case in Tanzania, where −14010*C was the only functional allele reported. We also found only this one functional allele in the Tanzanian Niger-Congo language speaking milk drinkers we tested (the Chagga). Interestingly this particular allele is present in the Masai who, like the Suri, speak a Nilo-Saharan language, but appears to have spread into the hunter-gatherers and also south-westwards to Southern Africa (Tishkoff et al. 2007; Breton et al. 2014; Macholdt et al. 2014a, b; Ranciaro et al. 2014).

Four of the five known functional alleles are at highest frequency in eastern Africa (Supplementary Figure 3) and it is possible that that this also reflects where they arose (East Africa or the Arabian peninsula), and importantly where the selection pressures have been the highest and maintained for the longest time. It is of interest that eastern Africa has more pastoralist groups than western Africa (Blench 1999). Although the reasons for this are unclear Blench (1999) has suggested that this may partly reflect displacement of indigenous pastoral groups in West Africa by the more recent Fulbe (Fulani), some time during the 1^st^ millennium A.D. Climatic differences may also have played a role, with bimodal rainfall patterns in East Africa supporting the development of pure pastoral systems better than in West Africa where the semi-arid zones with a single rainy season were more suited to agro-pastoralism (Blench 1999). Whatever the explanation, it is notable that the pastoralists in West Africa, Central Sahara and north western Africa who carry known functional LP enhancer alleles have alleles (−13910*T in the Fulani and the Berber (Mulcare et al. 2004; Myles et al. 2005) and −13907*G, −13910*T and −13915*G in the Shewa Arabs) that are likely the result of introgression from Europe, and East Africa. No other alleles have been found.

By comparing differences in diversity across the enhancer region in populations with a history of milk drinking with non-milk drinking groups, we also attempted to further refine the sequence region that is likely to have been important and identify other candidate loci. It can be seen from the sliding window approach that three small sections of the enhancer sequence account for most of the diversity, of which two show the most clear-cut differences between the milk drinkers and non-milk drinkers. While this clustering provides a pointer to defining the limits of the part of the enhancer that plays a role in preventing down-regulation of lactase after weaning, it is clear that not all variants have a functional effect. For example, −13913*C was shown to be more prevalent in non-digesters in our recent study (Jones et al. 2013). However the regions from −14005 to −13960 and −13895 to −13830, which revealed no allelic variants, seem unlikely to house elements relevant to down-regulation, but the fact that this sequence is also relatively conserved in primates (Jones et al. 2013) may indicate elements that play a role in other aspects of lactase expression.

Although no other strong functional candidate was identified, −13800*T is present in 24 % of the Nuer, a group who are cattle pastoralists and also cultivators, with a reported lactase persistence frequency of just 22 % (Bayoumi et al. 1982), making this allele worth consideration. However, our studies on Ethiopian digesters and non-digesters suggest that it is not functional since it was present in one non-digester, but absent from three Nuer who are digesters. Thus there is still a curious lack of functional alleles in the Nilo-Saharan Nuer, as well as a few other Ethiopian digesters (Jones et al. 2013). It is possible that in these people, and also in the Wolof (for which the literature suggests a frequency of lactase persistence phenotype of about 50 % in the specific group tested (Arnold et al. 1980)), there are nucleotide changes in sequence regions other than the enhancer, or that some kind of epigenetic adaptation occurs.

In summary, comparisons of the allele distributions highlight that these lactase persistence alleles are both regional and also to some extent linguistically partitioned, as previously observed for genome-wide data of Ethiopian groups (Pagani et al. 2012). The differences in diversity are also linguistically and regionally partitioned, but are not seen in the linked control regions tested emphasising that the effect is specific to the known functional enhancer. Diversity is greatest in East Africa where there is a longer tradition of pastoralism and declines further South and West where there are fewer pastoralist groups with more recent origin and less time over which selection could have operated. This distribution thus illustrates the effects of selection for lactase persistence in milk-drinking groups, set against the stochastic effects of the occurrence of the mutations and migration of the alleles, and contacts between the groups in which they arose, as well as the length of time over which selection has been operating.

## Electronic supplementary material

Supplementary material 1 (PDF 156 kb)

Supplementary material 2 (PDF 185 kb)

Supplementary material 3 (PDF 350 kb)

Supplementary material 4 (PDF 87 kb)

Supplementary material 5 (PDF 79 kb)

Supplementary material 6 (PDF 78 kb)
