# Supplementary material for: Diversity of lactase persistence in African milk drinkers
Source: Hum Genet. 2015 Jun 9;134(8):917–25. doi: 10.1007/s00439-015-1573-2 (PMC4495257; doi:10.1007/s00439-015-1573-2)
Supplement: Supplementary file 1 — Supplementary material 1 (PDF 156 kb) [file 439_2015_1573_MOESM1_ESM.pdf]

Supplementary Figure 1-

Sliding window plot of nucleotide diversity Pi, (Y axis) across the *LCT* enhancer.

Windows of 20 nucleotides with overlaps of 5 nucleotides. Nucleotide positions upstream of the start of transcription of *LCT* (X axis). Upper panel: non-milk drinkers and lower panel milk drinkers. See keys for groups. Note that many of the non-milk drinkers show no enhancer alleles at all (Supplementary Table 2) so that diversity is zero across the whole region and the lines cannot be seen.

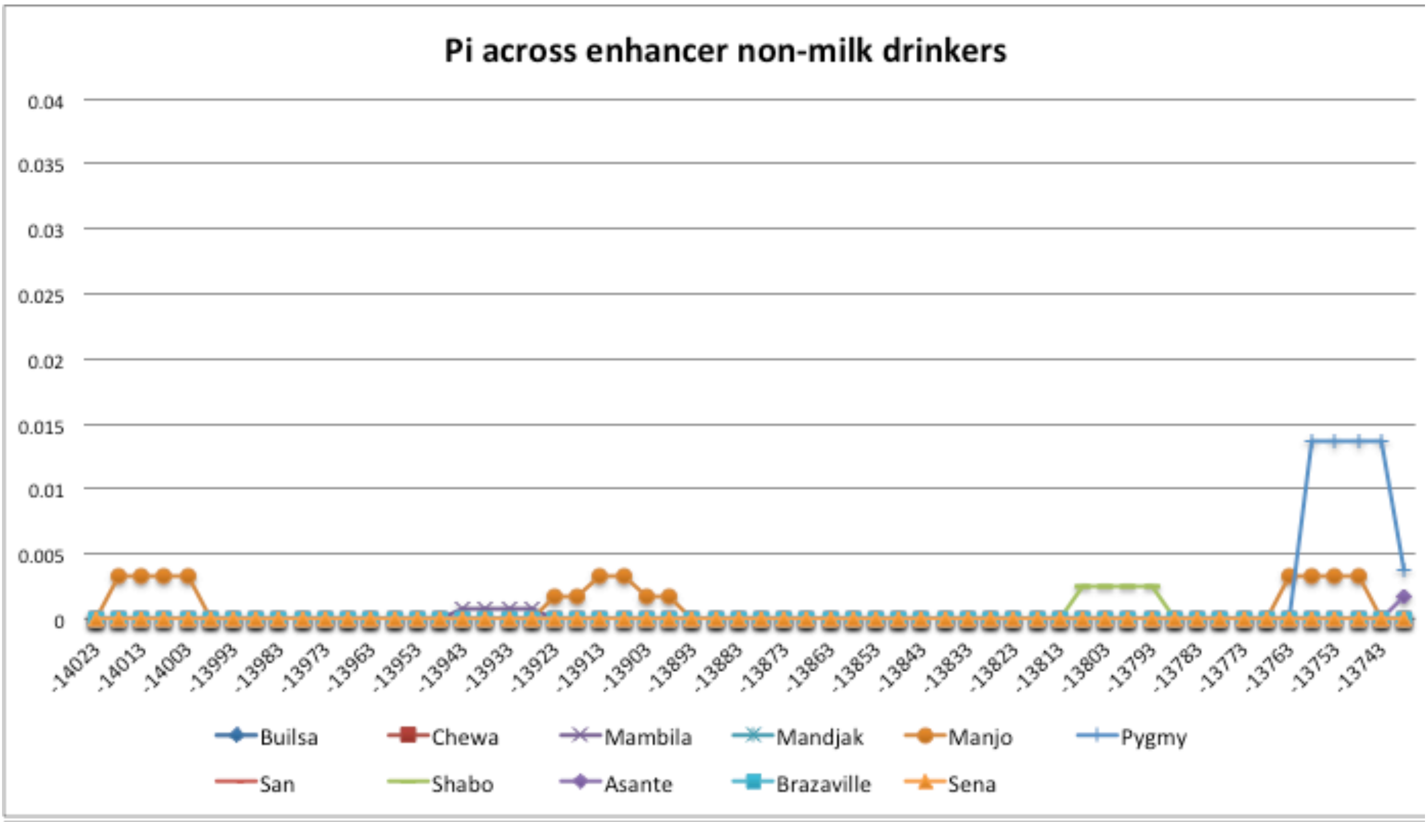
Pi across enhancer non-milk drinkers
0.04
0.035
0.03
0.025
0.02
0.015
0.01
0.005
0
-14023
-14013
-14003
-13993
-13983
-13973
-13963
-13953
-13943
-13933
-13923
-13913
-13903
-13893
-13883
-13873
-13863
-13853
-13843
-13833
-13823
-13813
-13803
-13793
-13783
-13773
-13763
-13753
-13743
Builsa
Chewa
Mambila
Mandjak
Manjo
Pygmy
San
Shabo
Asante
Brazaville
Sena

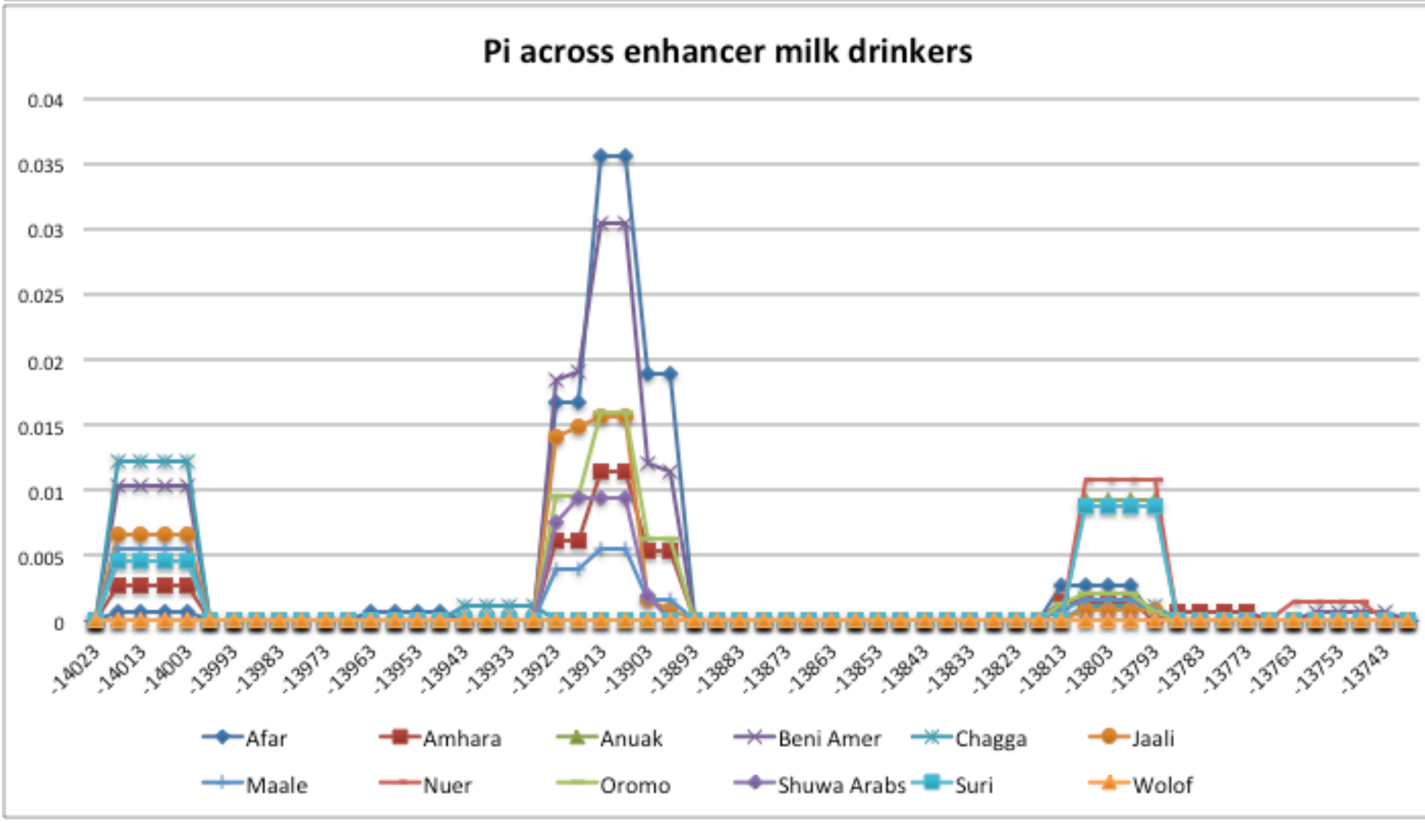
Pi across enhancer milk drinkers
0.04
0.035
0.03
0.025
0.02
0.015
0.01
0.005
0
-14023
-14013
-14003
-13993
-13983
-13973
-13963
-13953
-13943
-13933
-13923
-13913
-13903
-13893
-13883
-13873
-13863
-13853
-13843
-13833
-13823
-13813
-13803
-13793
-13783
-13773
-13763
-13753
-13743
Afar
Amhara
Anuak
Beni Amer
Chagga
Jaali
Maale
Nuer
Oromo
Shuwa Arabs
Suri
Wolof
