# Supplementary material for: Diversity of lactase persistence in African milk drinkers
Source: Hum Genet. 2015 Jun 9;134(8):917–25. doi: 10.1007/s00439-015-1573-2 (PMC4495257; doi:10.1007/s00439-015-1573-2)
Supplement: Supplementary file 2 — Supplementary material 2 (PDF 185 kb) [file 439_2015_1573_MOESM2_ESM.pdf]

Supplementary Figure 2. Correlation of LP frequency and nucleotide diversity. – extension of Figure 2. (all data (A) and data divided by language group and milk drinking status (B-D)) Only in the non-milk drinkers is there no significant correlation.

A

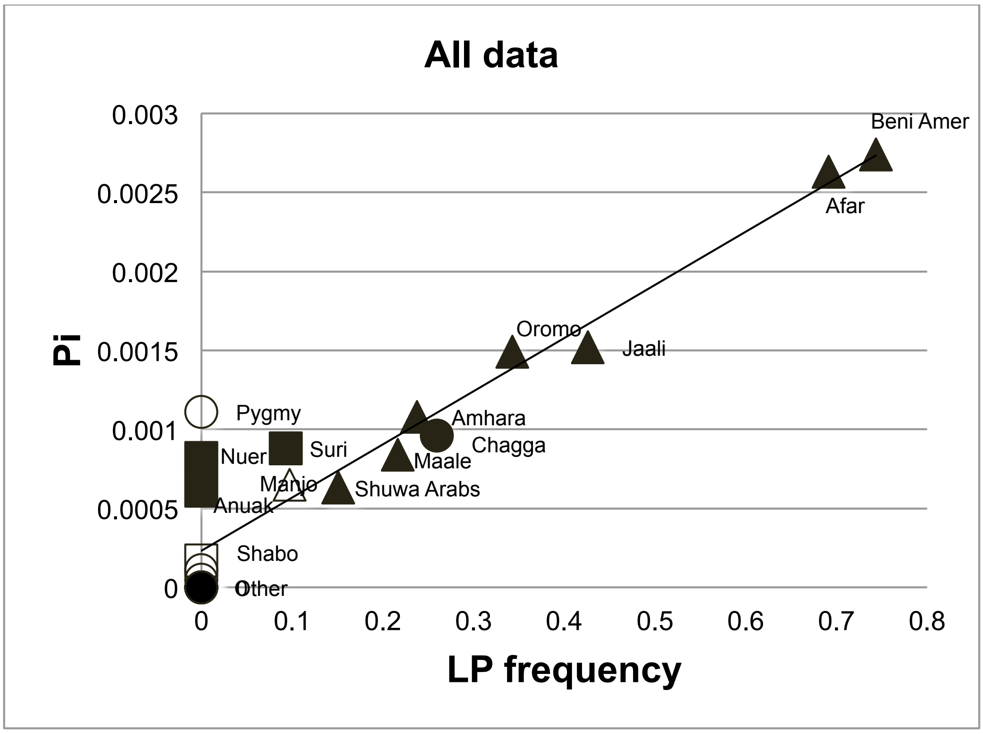

$r^2 = 0.86$ $p << 0.001$

B

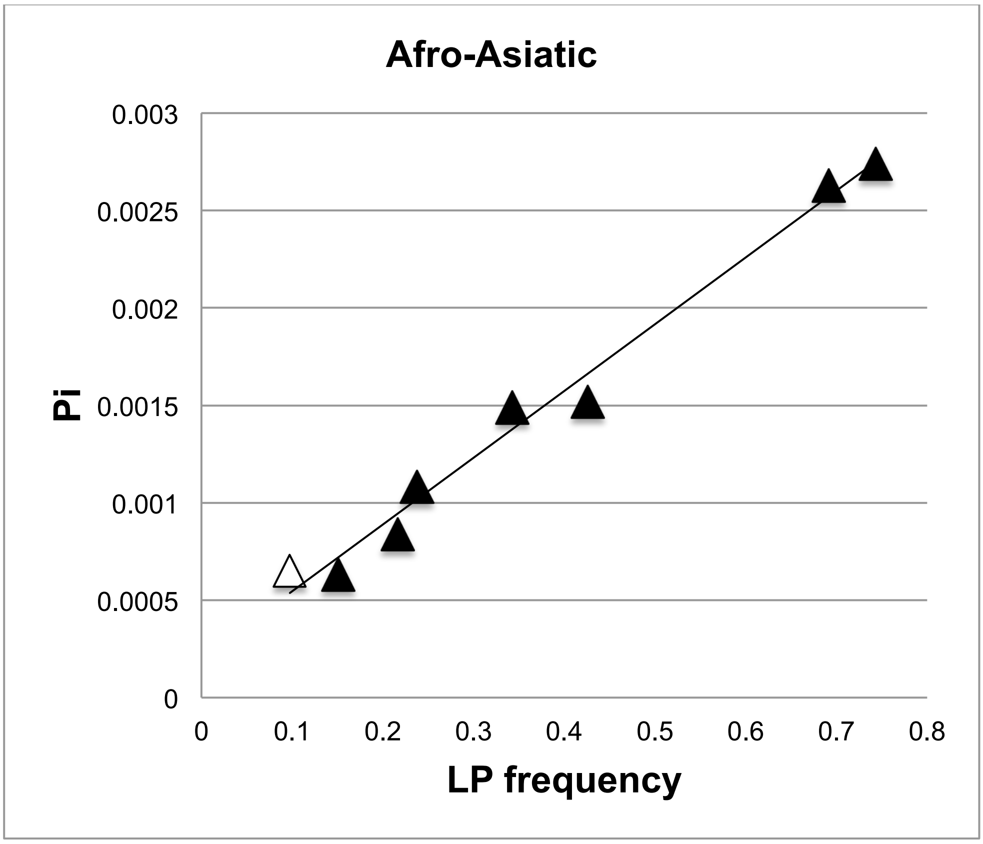

$r^2=0.99$ $p<0.01$

C

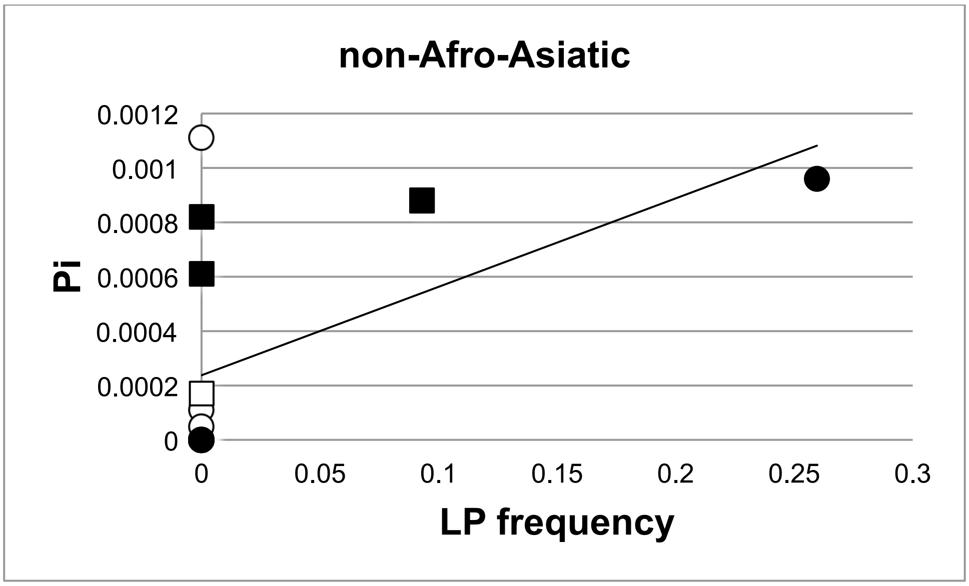

$r^2=0.28$ $p<0.05$

D

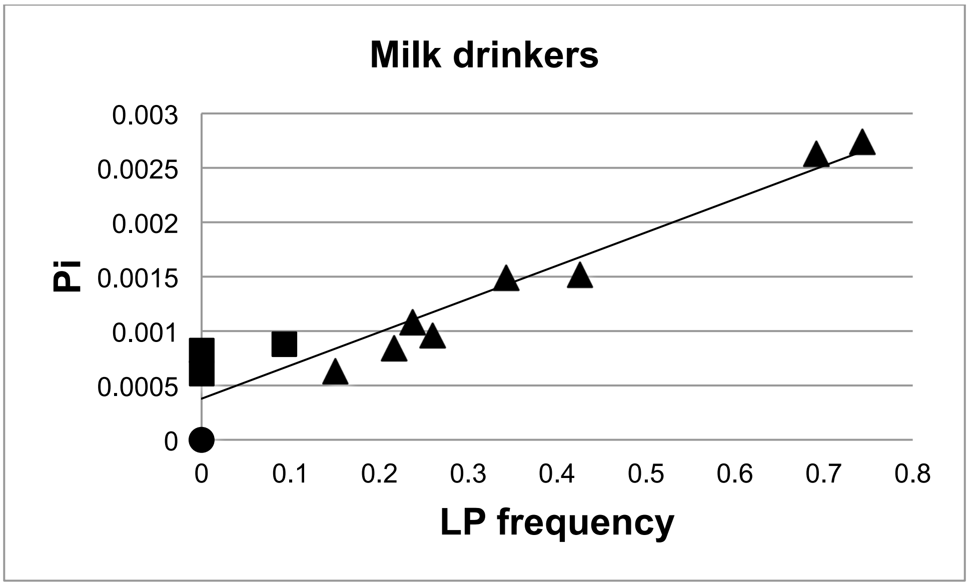

$r^2=0.91$ $p<0.001$

E

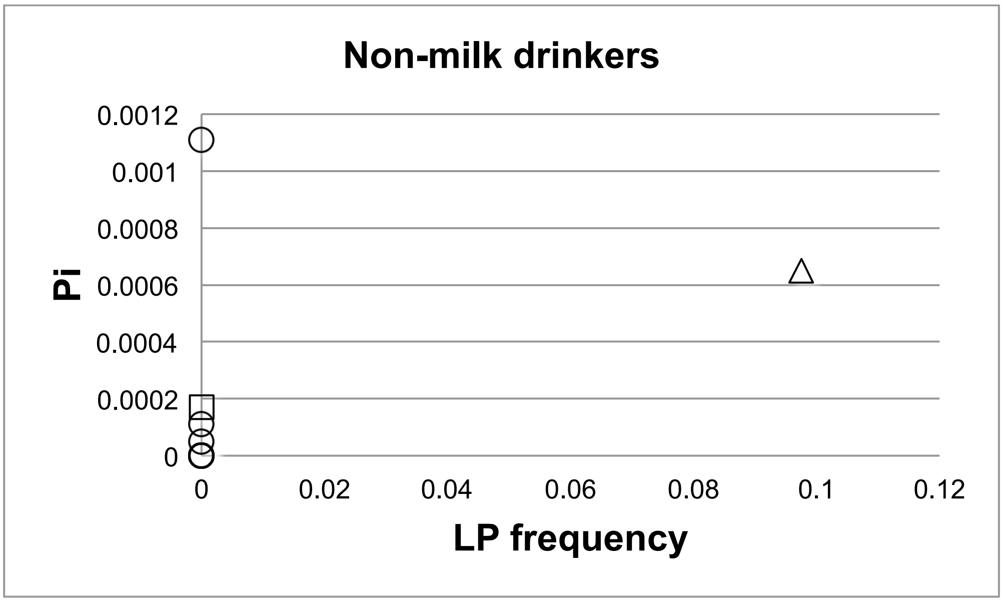

Non-milk drinkers
0.0012
0.001
0.0008
0.0006
0.0004
0.0002
0
Pi
0
0.02
0.04
0.06
0.08
0.1
0.12
LP frequency

r^2=0.19 ns
