# Supplementary material for: Diversity of lactase persistence in African milk drinkers
Source: Hum Genet. 2015 Jun 9;134(8):917–25. doi: 10.1007/s00439-015-1573-2 (PMC4495257; doi:10.1007/s00439-015-1573-2)

Supplementary Figure 3

Contour maps showing the distribution of the 4 most frequent individual LP functional enhancer alleles in comparison with the distribution of LP frequency calculated from all five functional alleles together. *-13910*T* is too rare to make a meaningful map. Note that for *-13907*G* particularly, the absence of data points in the Middle East in this study slightly distorts the contour map. Fuller maps will be shown in our next publication: Liebert et al in preparation.

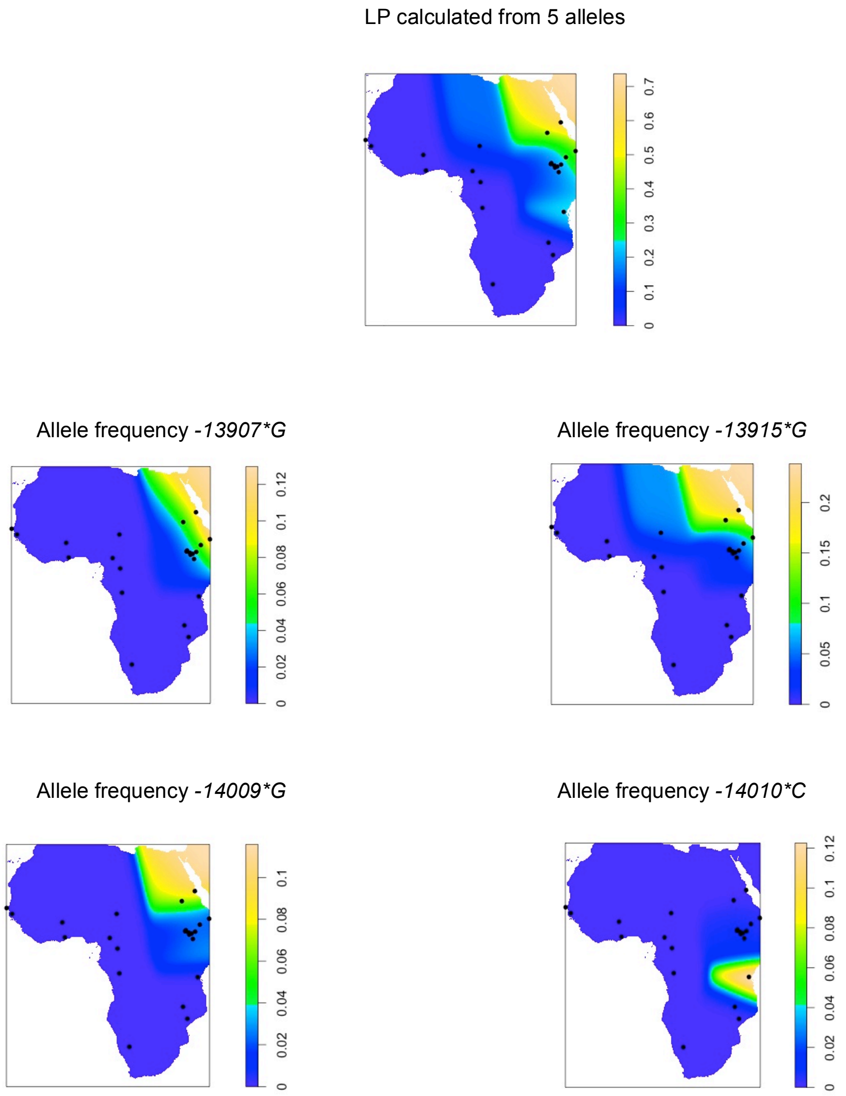

Supplement: Supplementary file 3 — Supplementary material 3 (PDF 350 kb) [file 439_2015_1573_MOESM3_ESM.pdf]
