# Supplementary material for: Diversity of lactase persistence in African milk drinkers
Source: Hum Genet. 2015 Jun 9;134(8):917–25. doi: 10.1007/s00439-015-1573-2 (PMC4495257; doi:10.1007/s00439-015-1573-2)
Supplement: Supplementary file 4 — Supplementary material 4 (PDF 87 kb) [file 439_2015_1573_MOESM4_ESM.pdf]

Country	Major language super -family	Language Group/Sub-group	Group desgnation	Murdock 1959 section ref	Milk drinking and Animal milked	Collection longitude	Collection latitude	n chromosomes	
**Main anlyses**									
Ghana	Niger-Congo	Volta-Congo	Asante	32:18	-	-0.55	6.17	68	
Congo	Niger-Congo	Narrow Bantu	Brazaville (Kongo+)	38:1	-	15.28	-4.27	90	
Ghana	Niger-Congo	Volta-Congo	Builsa	35:2	-	-1.29	10.53	38	
Malawi	Niger-Congo	Narrow Bantu	Chewa	38:47	-	33.78	-13.98	68	
Cameroon	Niger-Congo	Benue-Congo	Mambila	13:23	-	12.5	6	122	
Senegal	Niger-Congo	Atlantic	Mandjak	34:9	-	-15.88	12.99	92	
Ethiopia	Afro-Asiatic	Omotic	Manjo	10:4	-	36.23	7.27	60	
Cameroon	Niger-Congo	Narrow Bantu	Pygmy	8:1*	-	14.75	2.92	32	
Namibia	Khoisan		San	9	-	18.19	-25.6	22	
Mozambique	Niger-Congo	Narrow Bantu	Sena	38	-	35.05	-17.44	92	
Ethiopia	Nilo-Saharan/ unclassified &		Shabo	23:3*	-	35.41	7.56	40	
Ethiopia	Afro-Asiatic	Cushitic	Afar	41:1	Camels, cattle, goats, sheep	41.36	11.6	144	
Ethiopia	Afro-Asiatic	Semitic	Amhara	22:1	Cattle, goats, sheep	38.66	9.87	142	
Ethiopia	Nilo-Saharan		Anuak	21:1	Cattle, goats, sheep	34.41	7.95	138	
Sudan	Afro-Asiatic	Cushitic	Beni Amer	40:3	Camels, cattle, goats, sheep	37.22	19.62	154	
Tanzania	Niger-Congo	Narrow Bantu	Chagga	44:1	Cattle, goats, sheep	38.05	-5.38	86	
Sudan	Afro-Asiatic	Semitic	Jaali	54:6	Cattle	33.43	16.69	128	
Ethiopia	Afro-Asiatic	Omotic	Maale	23:7	Cattle, goats, sheep	36.64	5.71	122	
Ethiopia	Nilo-Saharan		Nuer	43:7	Cattle, goats, sheep	34.58	8.25	66	
Ethiopia	Afro-Asiatic	Cushitic	Oromo	42*	Cattle, goats, sheep	37.31	7.84	148	
Cameroon	Afro-Asiatic	Semitic	Shuwa Arabs	54:20	Cattle	14.5	13	102	
Ethiopia	Nilo-Saharan		Suri	43:4	Cattle, goats, sheep	35.59	7	84	
Senegal	Niger-Congo	Atlantic	Wolof	34:12	Cattle, goats, sheep	-17.45	14.69	84	
**Figure 4**									
Ethiopia	Afro-Asiatic	Cushitic	Southern Borana Oromo	42:3	Cattle, goats, sheep, Camel	39.05	3.54	208	furthest south
Ethiopia	Afro-Asiatic	Cushitic	Wallaga-Begi Oromo	42:8	Cattle	34.62	9.39	172	
Ethiopia	Afro-Asiatic	Cushitic	Harar Oromo	42*	Cattle	42.01	9.4	176	
Ethiopia	Afro-Asiatic	Cushitic	Salale Oromo	42*	Cattle	38.74	9.78	176	furthest north
Ethiopia	Afro-Asiatic	Cushitic	Oromo diverse	42*	Cattle	various	various	148	Jones et al 2013

Supplementary Table 1 Geographic locations of collection points, classification of first languages spoken and animal milking practise of group

Non-milk drinkers shown in grey   Approximate midpoints of group location taken where there were various or uncertain collection sites

Note that the precise spellings of the groups sometimes varied and those referred to by other older names or using very different spellings in Murdock, are marked with an *. Other sources were used to check correspondances

& the Shabo language originally classified as Nilo-Saharan has now been declassified: Schnobelen, T. 2009: Unclassifying Shabo: language documentation & linguistic theory 2. in Austen, P.K., Bond, O., Charette,M., Nathan, D. & Sellis, P. Proceedings of the Conference on Language Documentation and Linguistic theory, SOAS London.
