# Supplementary material for: Diversity of lactase persistence in African milk drinkers
Source: Hum Genet. 2015 Jun 9;134(8):917–25. doi: 10.1007/s00439-015-1573-2 (PMC4495257; doi:10.1007/s00439-015-1573-2)
Supplement: Supplementary file 5 — Supplementary material 5 (PDF 79 kb) [file 439_2015_1573_MOESM5_ESM.pdf]

Population	Asante	Brazaville	Builsa	Chewa	Mambila	Mandjak	Manjo	Pygmy	San	Sena	Shabo	Afar	Amhara	Anuak	BeniAmer	Chagga	Jaali	Maale	Nuer	Oromo	ShuwaArabs	Suri	Wolof	Grand Total
N chromosomes	68	90	38	68	122	92	60	32	22	92	40	144	142	138	154	86	128	122	66	148	102	84	84	2122
Milk drinkers	N	N	N	N	N	N	N	N	N	N	N	Y	Y	Y	Y	Y	Y	Y	Y	Y	Y	Y	Y	
**Control region 1**																								
G-30211T	0	0	0	0	0	0	0	0	0	0	0	0	0	0	0	0	0	0	0	0.007	0	0	0	0.000
G-30210C	0.206	0.078	0.105	0.118	0.098	0.174	0.350	0.094	0.136	0.098	0.125	0.333	0.507	0.304	0.201	0.349	0.250	0.434	0.212	0.459	0.284	0.417	0.226	0.271
Del-30203In	0	0.011	0	0	0	0	0	0	0	0	0	0	0	0	0.006	0	0	0	0	0	0	0.012	0	0.001
A-30196G	0	0	0	0	0	0	0	0	0	0	0.025	0	0	0.007	0	0	0	0	0.045	0	0	0	0	0.002
A-30182G	0.162	0.267	0.289	0.324	0.189	0.207	0.050	0.406	0.045	0.304	0.325	0.236	0.169	0.094	0.351	0.163	0.266	0.115	0.152	0.176	0.216	0.060	0.202	0.205
A-30160T	0	0	0	0	0	0	0	0	0	0	0.075	0	0	0	0	0	0	0	0.015	0	0	0	0	0.002
TC-30070/1AA	0	0	0	0.015	0	0	0	0	0	0	0	0	0	0.007	0.013	0	0	0	0	0	0	0	0	0.002
T-30069G	0	0.022	0	0	0	0	0	0	0	0	0	0	0	0	0	0	0	0	0	0	0	0	0	0.001
A-30036G	0	0	0	0	0	0	0	0	0	0	0	0	0	0	0	0	0	0.008	0	0	0	0	0	0.000
G-29949C	0.206	0.100	0.132	0.132	0.123	0.152	0.050	0.156	0	0.109	0.150	0.000	0.007	0.051	0.013	0.116	0.031	0.008	0.045	0.014	0.020	0.012	0.119	0.063
G-29942A	0	0	0	0	0	0	0	0	0.045	0	0	0	0	0	0	0	0	0	0	0	0	0	0	0.000
Control 1 sites	3	5	3	4	3	3	3	3	3	3	5	2	3	5	5	3	3	4	5	4	3	4	3	11
haplotypes	4	6	4	5	4	5	4	4	4	4	6	3	4	6	6	4	4	5	6	5	4	5	5	14
H	0.718	0.641	0.681	0.701	0.596	0.69	0.58	0.706	0.333	0.654	0.769	0.652	0.618	0.603	0.664	0.708	0.666	0.614	0.623	0.643	0.648	0.569	0.694	0.674
Pi	2.84E-03	2.38E-03	2.57E-03	2.77E-03	2.13E-03	2.67E-03	1.98E-03	2.86E-03	1.29E-03	2.42E-03	3.41E-03	2.45E-03	2.42E-03	2.19E-03	2.56E-03	2.85E-03	2.51E-03	2.21E-03	2.44E-03	2.51E-03	2.39E-03	1.97E-03	2.70E-03	2.59E-03
**Enhancer**																								
G-14010C	0	0	0	0	0	0	0	0	0	0	0	0	0	0	0	0.140	0	0	0	0.014	0	0.048	0	0.008
T-14009G	0	0	0	0	0	0	0.033	0	0	0	0	0.007	0.028	0	0.117	0	0.070	0.057	0	0.034	0	0	0	0.022
A-13957G	0	0	0	0	0	0	0	0	0	0	0	0.007	0	0	0	0	0	0	0	0	0	0	0	0.000
G-13937A	0	0	0	0	0.008	0	0	0	0	0	0	0	0	0	0	0.012	0	0	0	0	0	0	0	0.001
T-13915G	0	0	0	0	0	0	0	0	0	0	0	0.188	0.042	0	0.240	0	0.156	0.041	0	0.074	0.059	0	0	0.053
T-13913C	0	0	0	0	0	0	0.017	0	0	0	0	0.014	0.021	0	0	0	0.008	0	0	0.027	0.020	0	0	0.006
C-13910T	0	0	0	0	0	0	0	0	0	0	0	0	0	0	0.006	0	0.008	0	0	0	0.020	0	0	0.002
C-13907G	0	0	0	0	0	0	0.017	0	0	0	0	0.250	0.056	0	0.130	0	0.008	0.016	0	0.068	0	0	0	0.037
A-13806G	0	0	0	0	0	0	0	0	0	0	0	0.028	0.014	0	0	0	0	0.008	0	0.014	0	0	0	0.004
G-13800T	0	0	0	0	0	0	0	0	0	0	0.025	0	0	0.101	0	0.012	0.008	0.008	0.121	0.007	0	0.095	0	0.016
G-13779C	0	0	0	0	0	0	0	0	0	0	0	0	0.007	0	0	0	0	0	0	0	0	0	0	0.000
C-13753T	0	0	0	0	0	0	0.033	0	0	0	0	0	0	0	0	0	0	0	0.015	0	0	0	0	0.001
G-13752T	0	0	0	0	0	0	0	0.156	0	0	0	0	0	0	0.006	0	0	0	0	0	0	0	0	0.003
G-13732A	0.015	0	0	0	0	0	0	0.031	0	0	0	0	0	0	0	0	0	0	0	0	0	0	0	0.001
Enhancer sites	1	0	0	0	1	0	4	2	0	0	1	6	6	1	5	3	6	5	2	7	3	2	0	14
haplotypes	2	1	1	1	2	1	5	3	1	1	2	7	7	2	6	4	7	6	3	8	4	3	1	15
H	0.029	0	0	0	0.016	0	0.19	0.325	0	0	0.05	0.649	0.305	0.184	0.666	0.283	0.423	0.242	0.243	0.407	0.184	0.257	0	0.282
Pi	1.00E-04	0	0	0	5.00E-05	0	6.50E-04	1.11E-03	0	0	1.70E-04	2.63E-03	1.08E-03	6.10E-04	2.74E-03	9.60E-04	1.52E-03	8.40E-04	8.20E-04	1.49E-03	6.30E-04	8.80E-04	0	1.00E-03
LP allele	0	0	0	0	0	0	0.05	0	0	0	0	0.444	0.127	0	0.494	0.140	0.242	0.115	0	0.189	0.078	0.048	0	0.122
**Lp pheno**	0	0	0	0	0	0	0.0975	0	0	0	0	0.691	0.237	0	0.743	0.260	0.426	0.216	0	0.343	0.151	0.093	0	0.23
**Control region 2**																								
C-958T	0.088	0.033	0	0.015	0.008	0.087	0.250	0	0.045	0.022	0.025	0.306	0.430	0.087	0.136	0.093	0.219	0.295	0.045	0.378	0.167	0.262	0.143	0.169
A-946G	0	0	0	0	0	0	0.017	0	0	0	0	0	0	0	0	0	0	0	0	0	0	0	0	0.000
C-942G	0	0	0	0	0	0	0	0	0	0	0	0	0	0	0	0	0	0	0	0	0	0.048	0	0.002
TC-942/3ΔΔ	0.338	0.356	0.263	0.324	0.418	0.413	0.300	0.438	0.591	0.413	0.175	0.042	0.077	0.225	0.143	0.337	0.125	0.180	0.288	0.081	0.137	0.298	0.321	0.236
G-875A	0	0	0	0	0	0	0	0	0	0	0	0	0.007	0	0	0	0.023	0.008	0	0.020	0	0	0	0.004
C-814T	0	0	0	0.015	0	0	0	0	0	0	0	0	0	0	0	0	0	0	0	0	0	0	0	0.000
A-811G	0	0	0	0	0	0	0	0	0	0	0	0	0	0	0.013	0	0	0	0	0	0	0	0	0.001
C-766T	0	0	0	0	0	0	0	0	0	0.011	0	0	0	0	0	0	0	0	0	0	0	0	0	0.000
G-700C	0	0	0	0	0	0	0	0	0	0	0	0	0	0.014	0	0	0	0	0.030	0	0	0.012	0	0.002
A-694C	0	0	0	0	0	0	0.017	0	0	0	0	0	0	0	0	0	0	0	0	0	0	0	0	0.000
A-678G	0.162	0.267	0.289	0.338	0.197	0.185	0.050	0.406	0.045	0.293	0.325	0.236	0.176	0.087	0.364	0.163	0.297	0.115	0.061	0.169	0.167	0.060	0.250	0.204
Control sites	3	3	2	4	3	3	5	2	3	4	3	3	4	4	4	3	4	4	4	4	3	5	3	11
haplotypes	4	4	3	5	4	4	7	3	4	5	4	4	5	5	6	5	5	5	5	5	4	6	4	13
H	0.692	0.69	0.664	0.696	0.65	0.696	0.716	0.639	0.571	0.682	0.654	0.68	0.689	0.594	0.711	0.695	0.734	0.71	0.588	0.703	0.652	0.732	0.741	0.726
Pi	2.47E-03	2.56E-03	2.27E-03	2.65E-03	2.29E-03	2.65E-03	2.69E-03	2.79E-03	1.29E-03	2.70E-03	2.21E-03	2.41E-03	2.61E-03	1.94E-03	2.70E-03	2.49E-03	2.86E-03	2.60E-03	1.88E-03	2.62E-03	2.22E-03	2.89E-03	2.96E-03	2.73E-03

Supplementary Table 2. Allele frequencies and diversity measures for the three sequence regions analysed.
