# Supplementary material for: Diversity of lactase persistence in African milk drinkers
Source: Hum Genet. 2015 Jun 9;134(8):917–25. doi: 10.1007/s00439-015-1573-2 (PMC4495257; doi:10.1007/s00439-015-1573-2)
Supplement: Supplementary file 6 — Supplementary material 6 (PDF 78 kb) [file 439_2015_1573_MOESM6_ESM.pdf]

**Group**	N chromosomes	***-14010G>C***	***-14009T>G***	***-13957A>G***	***-13915T>G***	***-13913T>C***	***-13910C>T***	***-13907C>G***	***-13806A>G***	***-13800G>T***	***-13730T>G***	***LP****	**LP**	**Pi**	**H**
Southern Borana Oromo	208	0.048	0.010	0.010	0.221	0	0	0.077	0.034	0.010	0.024	0.356	0.590	2.49E-03	0.624
Wallaga-Begi Oromo	172	0	0.012	0.012	0.105	0.012	0	0.047	0.052	0	0.023	0.164	0.301	1.56E-03	0.425
Harar Oromo	176	0.017	0.006	0.006	0.125	0.006	0.011	0.102	0.028	0	0.028	0.261	0.454	2.01E-03	0.525
Salale Oromo	176	0.006	0.034	0.000	0.091	0.034	0	0.028	0.028	0	0.023	0.159	0.310	1.51E-03	0.410
Oromo students	216	0.014	0.019	0.009	0.106	0.019	0	0.056	0.014	0	0.042	0.195	0.352	1.70E-03	0.457

Supplementary Table 3

Supplementary Table for Figure 4 which shows enhancer allele frequencies and diversity data for the additional Oromo groups tested.
Known functional alleles highlighted in grey
